# Multimodal quantitative characterization and mechanistic exploration of paraspinal muscle groups in adolescent idiopathic scoliosis

**DOI:** 10.3389/fped.2026.1827163

**Published:** 2026-08-05

**Authors:** Ruibao Yang, ShangShang Ren, Jie Lin, Man Sun, Guangliang Jiang

**Affiliations:** 1 Department of Radiology, Orthopedics and Diabetes Hospital, Haikou City, China; 2 Out-Patient Department, Orthopedics and Diabetes Hospital, Haikou City, China

**Keywords:** adolescent idiopathic scoliosis, Cobb angle, fat fraction, MRI, paraspinal muscle

## Abstract

**Background:**

The mechanical properties of muscles and their interactions with three-dimensional spinal deformities are difficult to quantify with a single method. This study aimed to quantitatively characterize the paraspinal muscle groups in adolescent idiopathic scoliosis (AIS) using multimodal methods.

**Methods:**

The patients with AIS who received corrective surgery at our hospital from June 1, 2025, to August 31, 2025 were enrolled in this prospective study. Two independent evaluators used 3.0T magnetic resonance imaging (MRI) and digital radiography (DR) to analyze parameters in the bilateral paraspinal muscle groups.

**Results:**

This study enrolled 25 patients with AIS, including 15 cases with lumbar curvature and 10 with thoracic curvature. There were significant differences in the multimodal quantitative characterization of the convex and concave sides of scoliosis in AIS patients (*p* < 0.05). On the concave sides of the lumbar curvature, the erector spinae, multifidus muscles and psoas major muscles showed a higher fat fraction, higher signal intensity, and smaller volume than those on the convex sides. The erector spinae muscle was significantly thicker on the concave side in lumbar curvature, and on the convex side in thoracic curvature. Two evaluators showed good intra- and inter-observer consistency, with intraclass correlation coefficients (ICC) ranging from 0.75 to 0.85. The Cobb angle showed almost no correlation with the flexibility index on the concave side. For cases of thoracic curvature, the maximum vertebral inclination angle showed a strong positive correlation with the erector spinae thickness ratio on the convex side (*r* = 0.85).

**Conclusion:**

The quantitative assessment results of this exploratory study using MRI and DR methods showed that muscle asymmetry in the paraspinal muscles may interact with three-dimensional spinal deformity in patients with AIS.

## Introduction

As the most common spinal deformity in children and adolescents, adolescent idiopathic scoliosis (AIS) poses a serious threat to the health of millions of adolescents worldwide (1, 2). The global incidence rate of AIS ranges approximately from 0.5% to 4% (1, 2). Although genetic, biomechanical and neuromuscular factors are considered to contribute to the development of AIS, the exact pathogenesis has not been fully clarified to date (3–5). The imbalance of the paraspinal muscle groups may play an important role in the occurrence and progress of AIS. Multiple studies have found significant asymmetries in the muscle fiber types, electromyographic activity and histomorphological features of the paraspinal muscles between the concave and convex sides of patients with AIS (3, 6, 7). However, most existing studies have used only one single method, such as surface electromyography, CT, or ultrasound (3, 6, 7). The mechanical properties of muscles and their complex interactions with three-dimensional spinal deformities are difficult to quantify comprehensively through a single method.

Therefore, this study adopted multimodal assessments combining magnetic resonance imaging (MRI) and digital radiography (DR) to examine the paraspinal muscle groups in AIS patients. This study aimed to systematically analyze the imaging parameters of the paraspinal muscle groups and explore their potential mechanisms in the development and progression of AIS. This study focused on three key issues: ① comparing the differences in signal intensity, volume, fat fraction and maximum thickness of the bilateral paraspinal muscle groups; ② evaluating the consistency and feasibility of MRI and DR measurements between two evaluators using the intraclass correlation coefficient (ICC); ③ analyzing the correlations among the maximum vertebral inclination angle, COBB angle, fat fraction, flexibility index, and the paraspinal muscle groups. The results of this study may help provide a theoretical basis for individualized rehabilitation training, such as muscle strengthening, brace therapy and targeted exercise interventions.

## Patients and methods

### Patients

This study was conducted in compliance with the Declaration of Helsinki and was approved by the institutional ethics committee of our Hospital (No. 2025-Ethics Review-011). The parents of the adolescents enrolled in this study have signed written informed consents, and the data used were anonymous and unidentifiable. Patients with AIS who received corrective surgery at our hospital from June 1 to August 31, 2025 were continuously enrolled in this study.

Inclusion criteria were as follows: (1) aged 10–18 years; (2) AIS diagnosed by upright radiography of the entire spine in a standing position using DR; (3) the bones were not fully mature, with a Risser sign typically ≤ grade 4 using DR. (4) Cobb angle and flexibility index were measured using DR; (5) the volume, signal intensity, fat fraction, maximum thickness, and maximum vertebral inclination angle of the bilateral paraspinal muscle groups were measured using MRI.

Exclusion criteria were as follows: (1) Congenital spinal deformities, such as hemivertebrae and poor segmentation; (2) scoliosis caused by spinal deformities, malignant tumors, trauma, or other factors; (3) contraindications for magnetic resonance imaging; (4) incomplete or blurred images from either MRI or DR; (5) neuromuscular scoliosis, such as cerebral palsy, muscular dystrophy, or spinal cord injury; (6) syndromic scoliosis, such as Marfan syndrome or neurofibromatosis; (7) a history of spinal surgery; (8) presence of other serious systemic diseases that may affect the metabolism of the spine, muscles, or bones, including rheumatic and immune diseases, metabolic bone diseases, and severe osteoporosis.

### Equipment and parameters

MRI examinations were performed using a MAGNETOM Spectra 3T (Siemens AG, Munich, Germany). The scanning parameters for the T2 Dixon sequence were: TR **=** 3,500 ms, TE **=** 97 ms, slice thickness **=** 4 mm, slice spacing **=** 0.8 mm, and a field of view **=** 350 mm. For the T1-weighted sequence, the parameters were: TR **=** 995 milliseconds, TE **=** 10 milliseconds, slice thickness **=** 5 mm, slice spacing **=** 1 mm, and a field of view **=** 250 mm.

DR examinations were performed using a Multix Fusion Max (Siemens AG, Munich, Germany). The acquisition parameters for full-length spinal splicing were as follows: 90 kV in the upright position, with mAs automatically calculated based on patient thickness; 83 kV in the bending position, with mAs automatically calculated based on patient thickness.

### Image analysis and data measurement

**Figure 1:**
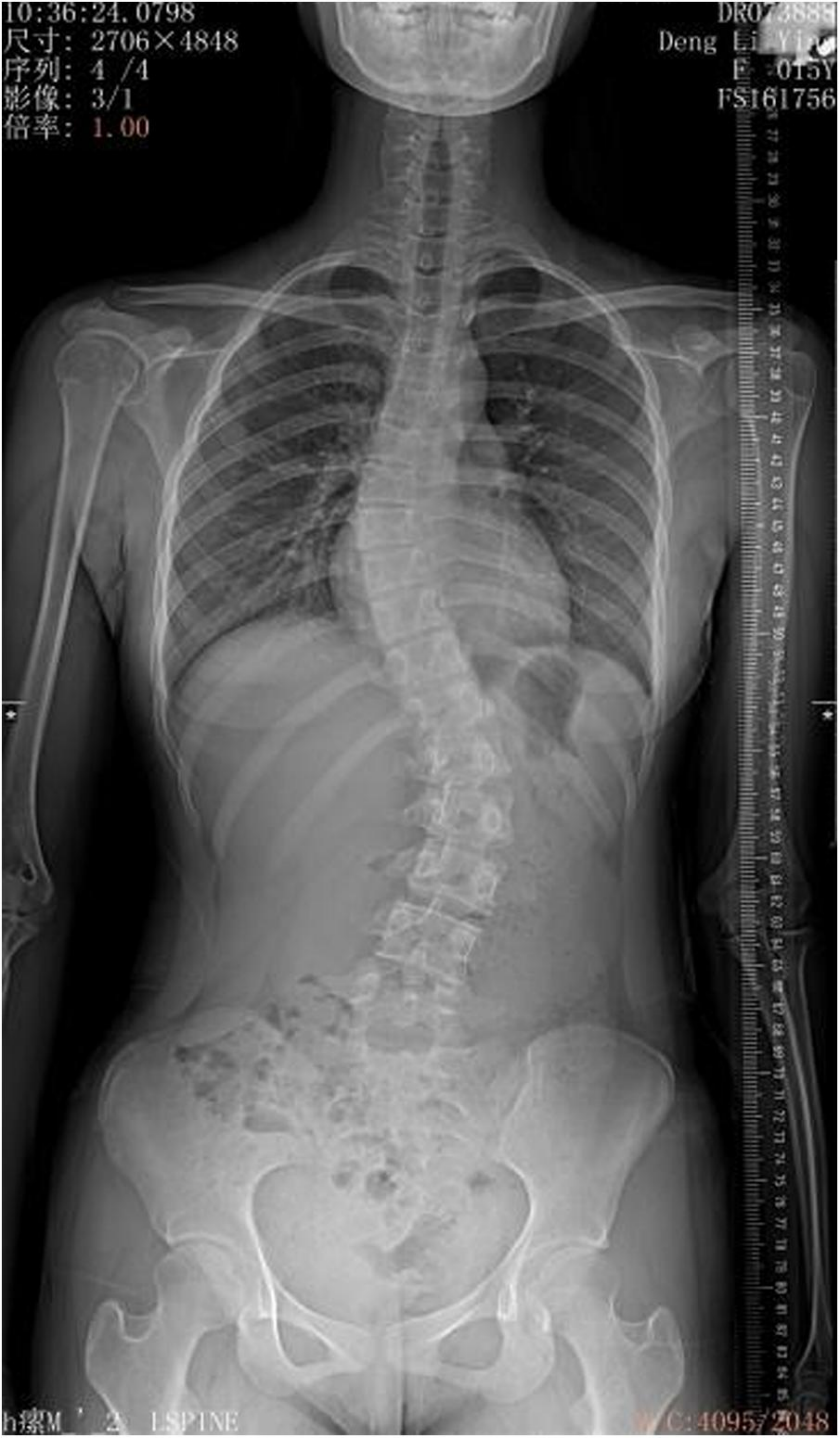
Cobb angle measurement vie DR. The Cobb angle was delineated between the extensions of the vertebral endplates at the upper and lower ends of the main scoliosis. The red dotted line represents the extension line of the upper vertebral endplate, and the yellow dotted line represents the extension line of the lower vertebral endplate.

DR was used to measure the following data. Cobb angle was defined as the angle formed by the extension lines of the vertebral endplates at the upper and lower ends of the main curvature on the anteroposterior x-ray of the entire spine (Figure 1).

Flexibility index was calculated as (*α*_1_–*α*_2_)/*α*_1_ × 100%. Here, *α*_1_ was the Cobb angle measured in the standard standing position, and *α*_2_ was the Cobb angle measured in the bending position. A negative value indicated that *α*1 was less than *α*2, while a positive value indicated that *α*1 was greater than *α*2. Both positive and negative values showed deviation from the normal growth direction of the spine. The flexibility index mainly measured the degree of deviation from the normal growth direction of the spine, ranging from 0% to 100%.

**Figure 2:**
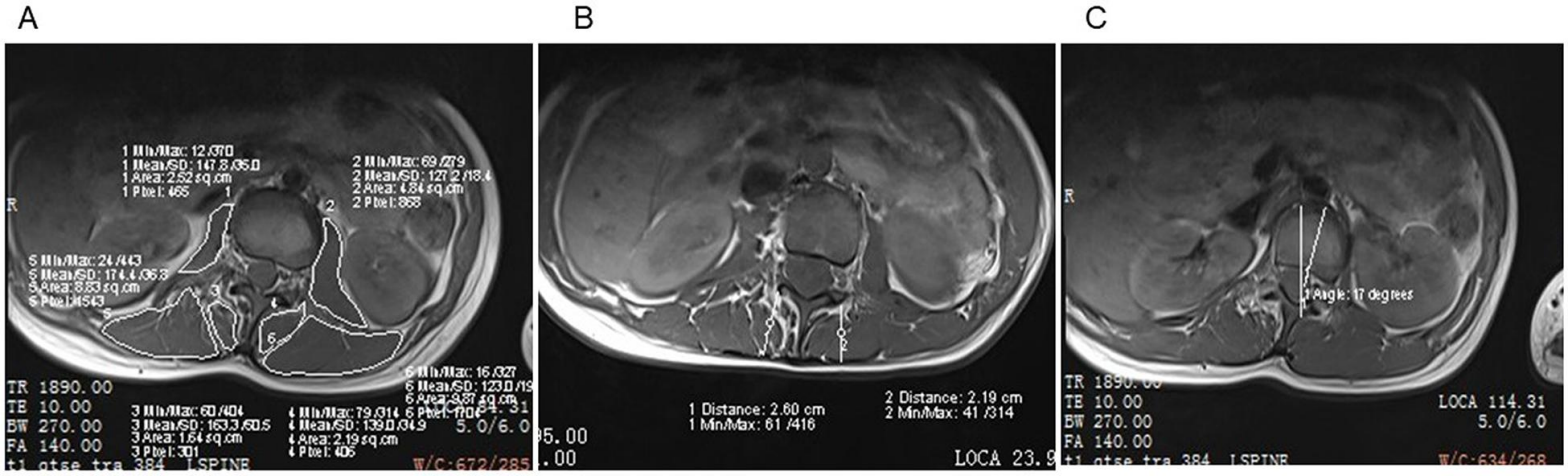
MRI examinations of different parameters. **(A)** Measurement of signal intensity and area of paraspinal muscle groups of the parietal vertebrae: Delineate the psoas major muscles (psoas major + quadratus lumborum), multifidus, and erector spinae muscles. The workstation automatically acquired the signal intensity and area. **(B)** The maximum thickness of the paraspinal muscle groups of the parietal vertebra. **(C)** The maximum vertebral inclination angle.

**Figure 3:**
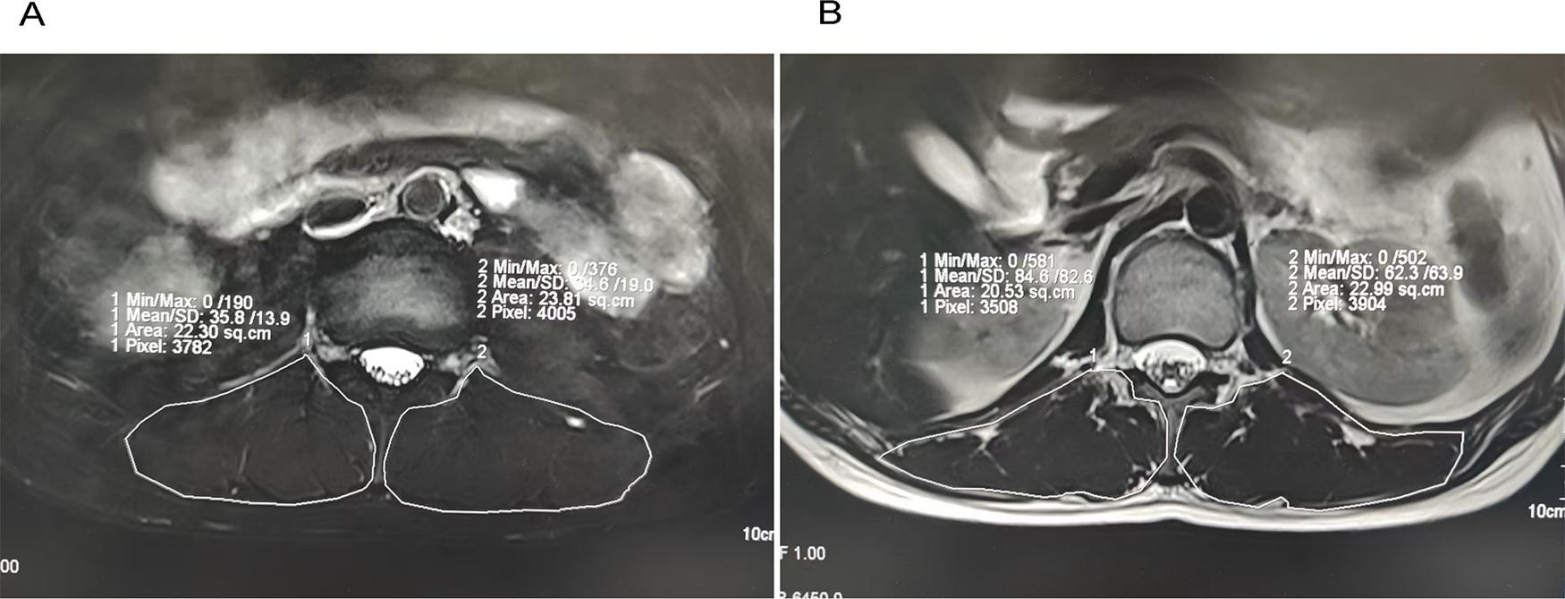
MRI examinations of fat fraction. **(A)** Signal intensity measurement on paraspinal muscle groups of the parietal vertebrae vie in sequence. **(B)** Signal values of the lipid sequence for the paraspinal muscle groups of the parietal vertebrae.

The following data of the paraspinal muscle groups were measured by MRI. The regions of interest (ROIs) of each paraspinal muscle group adjacent to the apex vertebra were delineated via T1 sequence (Figure 2A). The signal intensity was determined in the delineated regions. The area (S) of the regions was defined as the total area of all consecutive scanning slices. The volume was calculated by multiplying S by the slice thickness. The maximum thickness was defined as the maximum transverse diameter of each paraspinal muscle group adjacent to the apex vertebra, as seen on the T1 sequence (Figure 2B). The maximum vertebral inclination angle was defined as the angle between the apex vertebra in the coronal plane and the perpendicular line of its center using T1 sequence (Figure 2C). The fat fraction was calculated as 1 (signal values from the lipid sequence for the paraspinal muscle group adjacent to the apex vertebra/signal values from the “in” sequence of the same paraspinal muscle group) (Figures 3A,B). Two evaluators used standardized anatomical landmarks and drew the ROIs on the same slice for all patients.

### Statistical analysis

SPSS Statistics 26.0 version (IBM Corp., Armonk, NY, USA) was used for data analysis. A normality test was performed to determine whether the data conformed to a normal distribution. Paired Student's *t*-test was used to compare the measured data between the bilateral paraspinal muscle groups for variables following normal distribution, while the Wilcoxon rank sum test was applied for variables that did not. A *p*-value <0.05 was considered statistically significant. Inter- and intra-observer reliability was assessed using the intraclass correlation coefficient (ICC). An ICC value higher than 0.75 indicated good reliability, an ICC between 0.75 and 0.40 indicated moderate reliability, and an ICC lower than 0.40 meant poor reliability. Pearson correlation analysis was performed to evaluate the correlations among Cobb angle, fat fraction, flexibility index, and maximum vertebral inclination angle. The correlation strength was defined as poor if *R* ≤ 0.3, fair if 0.31 ≤ *R* ≤ 0.5, moderate if 0.51 ≤ *R* ≤ 0.6, moderately strong if 0.61 ≤ *R* ≤ 0.8, and very strong if *R* ≥ 0.81.

## Results

### The parameters of the paraspinal muscle groups

This prospective study enrolled 25 AIS patients, including 15 cases with lumbar curvature and 10 with thoracic curvature. These children included 19 girls and 6 boys, with a mean age of 14.35 ± 1.84 years and a body mass index (BMI) of 19.84 ± 3.72 kg/m^2^.

The parameters of the paraspinal muscle groups are shown in Table 1. There were significant differences in the muscle characteristics of the convex and concave sides in lumbar AIS patients (*P* < 0.05). The signal intensity of the psoas major muscles, multifidus, and erector spinae muscles on the concave side was significantly higher than that on the convex side. The volumes and fat fractions of these muscles on the concave side were also significantly smaller than those on the convex side (*P* < 0.05, Table 1).

**Table 1:** The parameters of the paraspinal muscle groups on the concave and convex sides in lumbar AIS patients.

Parameters	Signal intensity (Mean ± standard deviation)	Volume (Mean ± standard deviation)	Fat fraction (%)	*P*-value 1*	*P*-value 2^^^	*P*-value 3^&^
Concave side of lumbar AIS						
Psoas major muscle	142.2 ± 42.2	5.1 ± 2.2	23.3 ± 8.4	0.001	0.001	0.012
Multifidus muscles	157.8 ± 38.3	2.9 ± 1.1	30.8 ± 12.9	0.002	0.001	0.001
Erector spinae muscles	181.5 ± 52.6	17.3 ± 7.4	25.0 ± 10.0	0.001	0.001	0.002
Convex side of lumbar AIS						
Psoas major muscle	126.5 ± 37.6	7.4 ± 2.8	21.0 ± 10.0			
Multifidus muscles	137.6 ± 36.6	3.2 ± 1.1	25.0 ± 5.4			
Erector spinae muscles	134.0 ± 35.0	19.3 ± 8.6	23.0 ± 4.4			

Table 1 used paired student *t*-test.

*P*-value 1*: Signal intensity comparisons of the psoas major muscle, multifidus muscles and erector spinae muscles between concave and convex sides, and *P* < 0.05; *P*-value 2^^^: Volume comparisons of the psoas major muscle, multifidus muscles and erector spinae muscles between concave and convex sides, and *P* < 0.05; *P*-value 3^&^: Fat fraction comparisons of the psoas major muscle, multifidus muscles and erector spinae muscles between concave and convex sides, and *P* < 0.05.

The DR parameters of lumbar (*n* = 15) and thoracic (*n* = 10) AIS patients are shown in Table 2. The Cobb angle was 26.8 ± 9.7°, and the maximum vertebral inclination angle was 15.5 ± 6.8° in the lumbar AIS patients. The flexibility index was significantly lower on the convex side (−30.0 ± 24.0%) than on the concave side (69.6 ± 19.0%) (*P* = 0.001) among the lumbar AIS patients. The maximum thickness of the erector spinae muscles on the convex side was 2.87 ± 0.27 mm, which was higher than that on the concave side (2.4 ± 0.23 mm) (*P* = 0.001) in the lumbar AIS patients. The Cobb angle was 32.5 ± 13.7°, and the maximum vertebral inclination angle was 11.9 ± 5.2° in the thoracic AIS patients. The flexibility index of the convex side (−19.3 ± 48.0%) was significantly lower than on the concave side (63.2 ± 23.7%) (*P* = 0.001) in the thoracic AIS patients. The maximum thickness of the erector spinae muscles on the convex side was 2.7 ± 0.85 mm, which was higher than on the concave side (2.2 ± 0.83 mm) (*P* = 0.006) in the thoracic AIS patients.

**Table 2:** DR parameters of the lumbar (*n* = 15) and thoracic AIS (*n* = 10) patients.

Lumbar AIS (*n* = 15)	Cobb angle (°)	Maximum vertebral inclination angle (°)	Flexibility index of the convex side (%)	Flexibility index of the concave side (%)	Maximum thickness of the erector spinae muscles of the convex side (mm)	Maximum thickness of the erector spinae muscles of the concave side (mm)
Mean	26.8	15.5	−30	69.6	2.8	2.4
Standard deviation	9.7	6.8	24	19	0.27	0.23
*P* value*			0.001*		0.001*	

Thoracic AIS (*n* = 10)	Cobb angle (°)	Maximum vertebral inclination angle (°)	Flexibility index of the convex side (%)	Flexibility index of the concave side (%)	Maximum thickness of the erector spinae muscles of the convex side (mm)	Maximum thickness of the erector spinae muscles of the concave side (mm)
Mean	32.5	11.9	−19.3	63.2	2.7	2.2
Standard deviation	13.7	5.2	48.2	23.7	0.85	0.83
*P* value^			0.001^		0.006^	

*P* value*: *P* < 0.05, comparisons between the convex side and concave side in the lumbar AIS; *P* value^: *P* < 0.05, comparisons between the convex side and concave side in thoracic AIS.

### ICC analysis

The ICC values for the psoas major muscles, multifidus muscles and erector spinae muscles were compared between the concave and convex sides. The intra- and inter-observer ICC values ranged from 0.75 to 0.85, all of which were higher than 0.75, indicating good reliability (Table 3).

**Table 3:** ICC analysis of the two evaluators.

Values	Evaluator A ICC	Evaluator B ICC	ICC between A and B
Signal intensity (psoas major muscles)	0.84 (95% CI: 0.75, 0.92)	0.8 (95% CI: 0.62, 0.96)	0.82 (95% CI: 0.66, 0.92)
Signal intensity (multifidus muscles)	0.85 (95% CI: 0.73, 0.98)	0.82 (95% CI: 0.71, 0.94)	0.83 (95% CI: 0.76, 0.89)
Signal intensity (erector spinae muscles)	0.81 (95% CI: 0.06, 0.16)	0.84	0.82
Volume (psoas major muscles)	0.79 (95% CI: 0.66, 0.96)	0.82 (95% CI: 0.54, 0.92)	0.8 (95% CI: 0.66, 0.95)
Volume (multifidus muscles)	0.76 (95% CI: 0.72, 0.92)	0.8 (95% CI: 0.67, 0.94)	0.79 (95% CI: 0.61, 0.85)
Volume (erector spinae muscles)	0.82 (95% CI: 0.75, 0.92)	0.76 (95% CI: 0.62, 0.86)	0.81 (95% CI: 0.72, 0.86)
Cobb angle	0.78 (95% CI: 0.64, 0.96)	0.76 (95% CI: 0.71, 0.85)	0.76 (95% CI: 0.72, 0.89)
Maximum vertebral inclination angle	0.75 (95% CI: 0.61, 0.86)	0.81 (95% CI: 0.76, 0.89)	0.76 (95% CI: 0.68, 0.87)
Flexibility index	0.76 (95% CI: 0.71, 0.87)	0.82 (95% CI: 0.76, 0.92)	0.8 (95% CI: 0.72, 0.92)
Fat fraction	0.81 (95% CI: 0.72, 0.91)	0.85 (95% CI: 0.78, 0.95)	0.83 (95% CI: 0.76, 0.92)
Maximum thickness of erector spinae muscles	0.8 (95% CI: 0.72, 0.91)	0.81 (95% CI: 0.73, 0.89)	0.81 (95% CI: 0.68, 0.87)

ICC, intraclass correlation coefficient; CI, confidence interval.

### Pearson analysis

**Figure 4:**
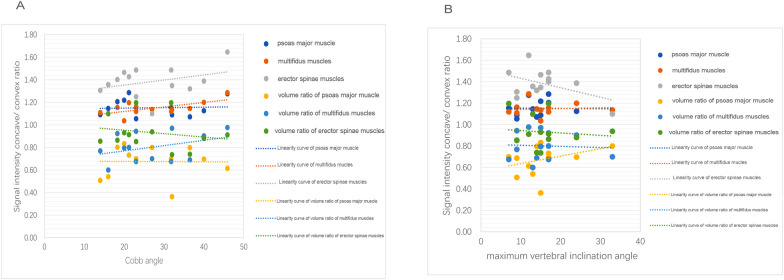
Pearson analysis results. **(A)** The scatter plot showing the correlation between the Cobb angle and the concave-to-convex ratios for psoas major, multifidus and erector spinae muscles in the lumbar region in lumbar AIS patients; **(B)** the scatter plot showing the correlation between the maximum vertebral inclination angle and the concave-to-convex ratios for the psoas major, multifidus, and erector spinae muscles in lumbar AIS patients.

Figure 4A shows the correlation analysis between the Cobb angle and the concave-to-convex ratios for psoas major, multifidus and erector spinae muscles in the lumbar region of patients with AIS. The concave-to-convex ratio of signal intensity was >1.0 and showed a weak positive correlation with the Cobb angle [*R* = 0.3 [95% CI: 0.26, 0.35], 0.16 [95% CI: 0.06, 0.25], 0.1 [95% CI: 0.06, 0.16], for the psoas major, multifidus, and erector spinae muscles, respectively]. The concave-to-convex volume ratio for the same muscles was <1.0 and showed a weak negative correlation with the Cobb angle [*R* = −0.02 [95% CI: −0.16, 0.18], −0.33 [95% CI: −0.39, −0.16], −0.2 [95% CI: −0.26, −0.11], respectively].

Figure 4B presents the correlation analyses between the maximum vertebral inclination angle and the concave-to-convex ratios for the psoas major, multifidus, and erector spinae muscles in lumbar AIS patients. The signal intensity ratio showed a weak positive correlation with the maximum vertebral inclination angle [*R* = 0.03 [95% CI: −0.02, 0.08], 0.04 [95% CI: 0.01, 0.07], and 0.01 [95% CI: 0.001, 0.002] for the psoas major, multifidus, and erector spinae muscles, respectively]. The volume ratio for the same muscles showed a weak negative correlation with the maximum vertebral inclination angle [*R* = −0.35 [95% CI: −0.35, −0.27], −0.23 [95% CI: −0.26, −0.18], and −0.39 [95% CI: −0.46, −0.30], respectively].

**Figure 5:**
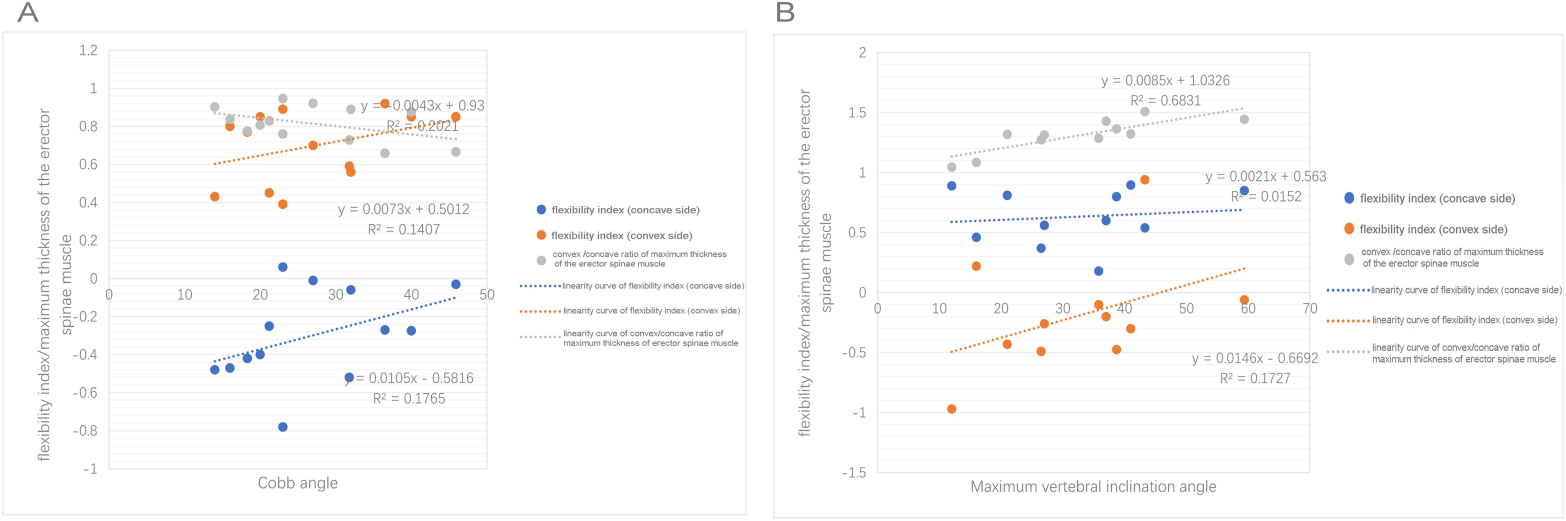
Pearson analysis results. **(A)** Scatter plot showing the correlations between Cobb angle, flexibility index, and the maximum thickness of the erector spinae muscle, respectively, in AIS lumbar patients; **(B)** scatter plot showing that correlation between the maximum vertebral inclination angle, flexibility index, and the maximum thickness of the erector spinae muscle, respectively, in AIS thoracic patients.

Figure 5A shows the correlations between the Cobb angle and the flexibility index, as well as the maximum thickness of the erector spinae muscle, respectively, in lumbar AIS patients. The correlation coefficient between the Cobb angle and the flexibility index on the convex side was 0.46 (95% CI: 0.4, 0.58), suggesting a moderate positive correlation; the correlation coefficient with the flexibility index on the concave side was −0.07 (95% CI: −0.15, −0.02), indicating a negligible correlation. The correlation coefficient between the Cobb angle and the concave-to-convex maximum thickness ratio of the erector spinae muscle was −0.45 (95% CI: −0.5, −0.42), suggesting a moderate negative correlation.

Figure 5B shows that correlations between the maximum vertebral inclination angle and the flexibility index, as well as concave-to-convex ratio of maximum thickness of the erector spinae muscle in thoracic AIS patients. The correlation coefficient between the maximum vertebral inclination angle and the flexibility index on the convex side was 0.42 (95% CI: 0.34, 0.49), indicating a moderate positive correlation. However, the correlation coefficient with the flexibility index on the concave side was 0.13 (95% CI: 0.1, 0.18), suggesting a weak positive correlation. The correlation coefficient between the maximum vertebral inclination angle and the maximum thickness convex/concave ratio of the erector spinae muscle was 0.85 (95% CI: 0.72, 0.95), suggesting a strong positive correlation.

## Discussion

The key findings of this study are as follows: ① the muscle signal intensity and fat fraction were significantly higher on the concave side than on the convex side, while the volume was lower on the concave side than on the convex side. In patients with lumbar AIS, the erector spinae muscle was thinner on the convex side than on the concave side, while in thoracic AIS patients, it was thicker on the convex side than on the concave side. ② the measurements of anatomical parameters of the paraspinal muscle group in these patients were highly reliable, with a high ICC indicating good consistency. ③ for lumbar AIS patients, the Cobb angle showed a moderate positive correlation with the flexibility index on the convex side, but no significant correlation with the flexibility index on the concave side. It also had a moderate positive correlation with the ratio of convex/concave thickness of the erector spinae muscle. For thoracic AIS patients, the maximum vertebral inclination angle showed a strong positive correlation with the flexibility index on the convex side, and the convex/concave ratio of the erector spinae muscle thickness, but only a weak positive correlation with the flexibility index on the concave side.

This study found that the paraspinal muscle groups in lumbar AIS patients showed significant bilateral asymmetry when quantitatively characterized by multimodal methods. This result is consistent with the results of previous studies (3, 6, 7). Previous research has also shown that there are significant differences in cell activity and histological grade between the convex and concave sides, which may affect the growth of muscles on both sides (8). In addition, the genetic causes of AIS may also influence muscle growth (9). The muscles on the concave side have been in a passive shortening state for a long time and have lost muscle fibers due to the constant mechanical loading from three-dimensional deformity (10–13). When abnormal stress loads occur, the muscle fibers on the convex side increase significantly in size. On the concave side, reduced muscle activity and muscle atrophy lead to increased fat infiltration. As a result, the signal intensity and fat fraction on the concave side were also significantly higher than on the convex side (14), which was validated by the results of the current study. Therefore, the function of these muscles degenerates along with energy metabolism disorders, resulting in both increased volume and increased signal intensity (10–13).

Larger muscle fiber asymmetry is closely related to greater curvature of the scoliosis (10, 11), and the Cobb angle is also closely related to muscle fibers properties, volume, the duration of scoliosis, and the degree of fat infiltration (10–12). The long-term progression of scoliosis may cause increased tension in the psoas major muscle on the affected side and weakening on the opposite side. This situation may result in a mechanical imbalance of the trunk and pelvis, which may further exacerbate the spinal curvature in AIS. A study on adult degenerative scoliosis of the spine found that a biomarker named “hsa_circ_0006156” may participate in the regulation lipid metabolism and may help to reduce fat deposition (15). However, there is limited research on molecular mechanism of AIS, and further investigations are warranted.

Nevertheless, the erector spinae muscle still maintains certain functional capacity, such as increasing the number and cross-sectional area of muscle fibers, to counteract abnormal stress (16). Notably, this study found that the thickness of the erector spinae muscles in lumbar and thoracic AIS patients showed opposite bilateral differences. We speculate (11–17) that, in AIS lumbar patients, the erector spinae muscles on the convex side may be prone to centrifugal injury resulting from continuous traction due to the large range of motion of the lumbar vertebrae and the weight they bear. This situation may result in less thickness on the convex side than on the concave side. In thoracic AIS patients, the thoracic cage has structural limitations and respiratory functional demands, and therefore the erector spinae muscles on the convex side must counteract the lateral stress on the ribs. Long-term high-tension stimulation in the thoracic section leads to compensatory hypertrophy, making the thickness of the convex side greater than on the concave side. This difference reflects the morphological specialization of different spinal segments to adapt to their respective biomechanical challenges, which might reveal the regional specific compensatory mechanisms in the pathological process of AIS progression.

This study showed that the intra- and inter-observer ICC values ranged from 0.75 to 0.85, indicating high reliability and reproducibility of the MRI and DR measurements. These results not only demonstrated the reliability of quantitative analysis using MRI and DR images, but also provided supporting data for standardized evaluation of these parameters in AIS patients.

As a core deformity parameter, the Cobb angle showed a strong positive correlation with the maximum vertebral inclination angle, suggesting that rotational deformity of the spine may progress synchronously with coronal scoliosis. The flexibility index of the convex side was moderately negatively correlated with the Cobb angle, which indicates that the ribs and soft tissue on the convex side gradually lose their elasticity due to continuous traction, forming a trend of “rigidity”. However, the flexibility index of the concave side showed little linear correlation with the Cobb angle, indicating that the muscles on the concave side may maintain some flexibility through compensatory remodeling. The convex/concave ratio of the erector spinae muscle thickness showed a strong positive correlation with the Cobb angle, suggesting that the imbalance of muscle groups on both sides is an important biomechanical factor for the progression of scoliosis (3, 6, 7, 17–19). This multi-dimensional correlation model revealed that the pathological process of AIS may involve not only geometric deformation of the skeletal structure, but also adaptive remodeling of surrounding soft tissues, particularly the muscular system. The progressive rigidity of the muscles on the convex side, along with progressive atrophy of the muscles on the concave side, collectively influences the dynamic evolution of spinal deformities (10–13). The variations in correlation among different indicators also suggested that, in addition to mechanical stress, factors such as neural regulation, metabolic microenvironment, and genetic susceptibility may play key roles at specific stages of AIS development (10–13). The molecular mechanisms warrant further investigation through multimodal imaging in combination with biomarker detection in future studies.

The limitations of this study are as follows. Firstly, the sample size is small and healthy controls are absent. This limitation makes it difficult to distinguish between pathological changes and normal physiological development. Secondly, although MRI quantitative analysis is fairly consistent within observers (ICC = 0.75–0.85), its ability to detect early fat infiltration and microstructural changes is limited by the scanning sequence parameters. Thirdly, manually delineating the ROIs may introduce subjective bias. Using a raw calculation method to assess muscle volume in three-dimensional space may also introduce bias. It is necessary to combine artificial intelligence-assisted segmentation technology to enhance objectivity. Fourth, the current conclusions are derived from the changes in imaging morphology, but they lack direct evidence from histological biopsy or molecular biology, such as the proportion of muscle fiber types and the expression of inflammatory factors, to verify the specific mechanisms of metabolic stress and steatosis. Fifth, the study focuses on the associations among the imaging parameters, but it did not establish a direct link between changes in muscle shape and symptom severity, brace effectiveness, or surgical outcomes. Finally, the multiple comparisons without correction for multiple testing may introduce bias into the results. Future prospective large-scale clinical trials are needed to expand the sample size, include a healthy control group, and apply more rigorous statistical methods in a multi-center setting to verify these findings.

## Conclusion

This study used multimodal quantitative analysis and found that the paraspinal muscle groups in AIS patients showed significant bilateral asymmetry. This asymmetry manifested as increased signal intensity and fat infiltration in the concave muscles, as well as a decreased volume, compared with the convex-side muscles. Moreover, in patients with lumbar and thoracic curvature, the erector spinae muscles exhibited opposite thickness distribution. These characteristics may reflect segment-specific adaptation to their respective biomechanical environment. The Cobb angle in lumbar AIS was positively correlated with signal intensity and fat fraction, and negatively correlated with volume. These findings suggest that direct measurements of morphological indicators may be insufficient to accurately predict AIS deformity progression. The correlations among imaging parameters in thoracic AIS patients were stronger, suggesting that the pathological mechanism in these patients may depend more heavily on soft tissue factors. This study provides exploratory evidence for muscle-targeted interventions in AIS. Future studies should combine functional MRI, electromyography, and molecular biological approaches to elucidate the neuromuscular regulatory network underlying paraspinal muscle dysfunction in AIS patients.

## Data Availability

The raw data supporting the conclusions of this article will be made available by the authors, without undue reservation.
